# Microbial Inactivation in the Liquid Phase Induced by Multigas Plasma Jet

**DOI:** 10.1371/journal.pone.0132381

**Published:** 2015-07-14

**Authors:** Toshihiro Takamatsu, Kodai Uehara, Yota Sasaki, Miyahara Hidekazu, Yuriko Matsumura, Atsuo Iwasawa, Norihiko Ito, Masahiro Kohno, Takeshi Azuma, Akitoshi Okino

**Affiliations:** 1 Department of Gastroenterology, Kobe University, Kobe, Japan; 2 Department of Energy Sciences, Tokyo Institute of Technology, Yokohama, Japan; 3 Department of Bioengineering, Tokyo Institute of Technology, Yokohama, Japan; 4 Veterinary Medical center, Tottori University, Tottori, Japan; University Paul Sabatier, FRANCE

## Abstract

Various gas atmospheric nonthermal plasmas were generated using a multigas plasma jet to treat microbial suspensions. Results indicated that carbon dioxide and nitrogen plasma had high sterilization effects. Carbon dioxide plasma, which generated the greatest amount of singlet oxygen than other gas plasmas, killed general bacteria and some fungi. On the other hand, nitrogen plasma, which generated the largest amount of OH radical, killed ≥6 log of 11 species of microorganisms, including general bacteria, fungi, acid-fast bacteria, spores, and viruses in 1–15 min. To identify reactive species responsible for bacterial inactivation, antioxidants were added to bacterial suspensions, which revealed that singlet oxygen and OH radicals had greatest inactivation effects.

## Introduction

The use of atmospheric nonthermal plasma has attracted attention as a novel method to inactivate bacteria on the surface of fresh foods and medical devices [1]. The plasma inactivation offers several benefits to bacterial inactivation than conventional methods, such as low temperature, low toxicity, high efficiency, and low cost [2]. Therefore, the bacterial inactivation effects of several recently developed plasma sources have been further investigated [3]. Current studies have reported that reactive species generated by plasma sources largely contribute to the inactivation of bacteria [4], [5], [6]. In particular, atomic oxygen and reactive oxygen species (ROS) have been identified as factors that contribute to bacterial inactivation because ROS are known to damage the cell walls, cell membranes, and DNA of microorganisms [7], [8]. However, details of the relationship between reactive species and bacterial inactivation remain unclear because the abilities of conventional plasma sources to generate gas species are limited: they can only generate particular gas species such as mock air plasma, helium plasma or argon plasma, which are relatively easy to generate [3]. To generate other gas plasma, it is required that the parameters of plasma source such as voltage, frequency, flow rate and plasma generation method are optimized depending on gas species. The kinds of reactive species and the amount vary greatly depending on the parameters [9]. Therefore, it is difficult to consider the bacteria inactivation relevant to plasma gas species compared with previous works used other plasma sources. However, there is no report to investigate bacterial inactivation by various gas plasmas with a device.

Our group successfully developed a multigas plasma jet, which can generate plasma from various gas species [10], [11], [12], [13].

The plasma jet can generate stable nonthermal plasma with various gases, including helium, argon, oxygen, and nitrogen, as well as mixtures of these gases. Thus, various reactive species, depending on the gas, can be produced. The aims of the present study were to identify effective reactive species for bacterial inactivation and the range of microorganisms susceptible to inactivation, as well as to assess the general effects of various reactive species on bacteria, viruses, fungi, acid-fast bacilli, and bacterial spores in order of tolerance.

## Materials and Methods

### Multigas plasma jet source

Experiments were conducted using a multigas plasma jet source, as described elsewhere [10], [11], [12], [13]. The body (83 mm in length) of the apparatus was grounded and an interior high voltage electrode was connected to an AC power supply (Plasma Concept Tokyo, Inc., Tokyo, Japan) to generate 16 kHz and 9 kV at approximately 10 W. The generated plasma flowed out through a 1 mm outlet at a rate of 1 L/min. This system can generate stable atmospheric plasma with various gas species, including argon, oxygen, nitrogen, carbon dioxide, mock air, and mixtures of these gases at low temperatures. For this study, a temperature-control unit was installed before a discharge unit for the preliminary control of the gas temperature. A gas supplied from a gas cylinder was cooled using a gas-cooling device that uses liquid nitrogen (-196°C), after which the gas was heated to a desired temperature using a heater to generate the plasma. Information of the plasma gas temperature was fed back to the heater, and the plasma gas temperature was fixed at 20°C [14]. The setup parameters of the plasma source are shown in Table 1.

**Table 1:** Setup parameter of plasma source.

Gas species	Ar, O_2_, N_2_, CO_2_, mock air
Gas flow rate	1 L/min
Gas temperature	20°C
Input power	10 W

Here we evaluated the effect of five plasma gas species on growth inhibition of *Staphylococcus aureus* and *Pseudomonas aeruginosa* (*n* = 3). After establishing effective bacterial inactivation conditions, plasma treatments against several microorganisms were performed.

### Bacterial inactivation using the multigas plasma jet

**Fig 1:**
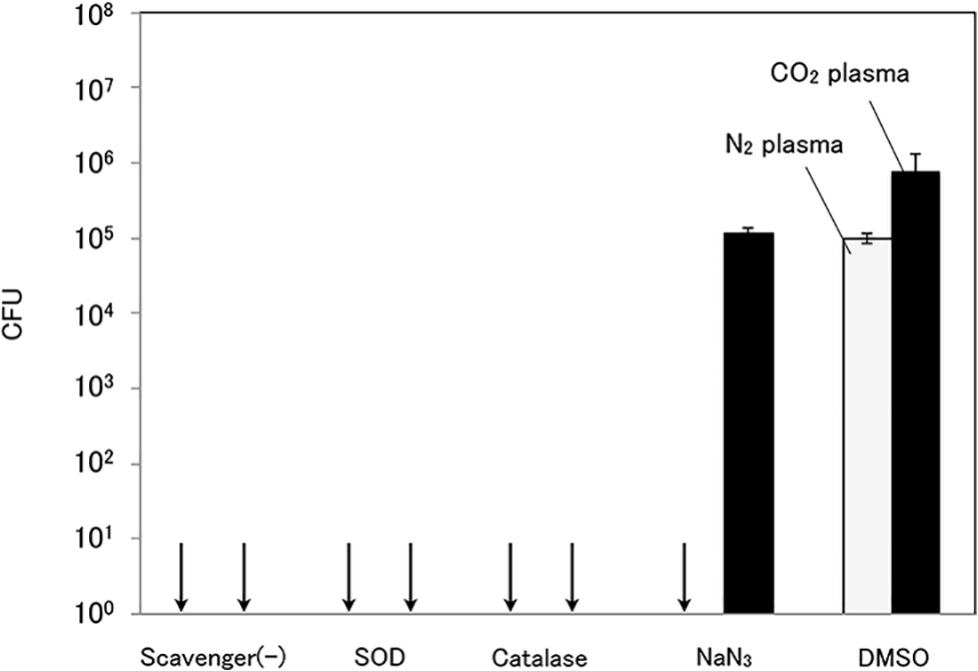
A schematic of the plasma treatment system using a multigas plasma jet source.

The microbial inactivation effects of our multigas plasma jet were investigated using several species of bacteria and fungi grown on different mediums at set incubation times and specific incubation temperatures, as shown in Table 2. The experimental species included the general bacteria *Staphylococcus aureus*, *Enterococcus faecalis*, *Escherichia coli*, *P*. *aeruginosa*, and methicillin-resistant *S*. *aureus* (MRSA), the acid-fast bacilli *Mycobacterium terrae* and *Mycobacterium abscessus*, the spore-producing bacteria *Bacillus cereus*, and the fungi *Candida albicans*, *Aspergillus niger*, and *Trichophyton mentagrophytes*. The bacteria and fungi were incubated on agar medium, and the colonies were harvested and suspended in phosphate-buffered saline (PBS) (−) (Nissui Pharmaceutical, Co. Ltd., Tokyo, Japan), with a pH value of 7.3–7.5. To prepare a pure spore suspension, *B*. *cereus* was incubated at 37°C for 7 days and then heat-treated at 50–60°C. Furthermore, the harvested spores were suspended in PBS(−) and stored at 4°C for further use. A schematic of the plasma treatment system is shown in Fig 1. The population of bacterial suspensions was fixed at >10^6^ cells in 200 μl of PBS(−) by McFarland standards. The plasma jet outlet was fixed at a distance of 6 mm from the surface of the bacterial suspension. After plasma treatment in open air, the bacterial suspensions were serially diluted by 10-fold and each dilution was incubated on agar medium. After incubation at the specified times, the number of colony forming units (CFU) was determined (*n* = 2–3).

**Table 2:** Target microorganisms.

	Microorganism	Strain	Agar plate	Incubation time,temperature
Bacteria	*S*. *aureus*	ATCC25923	Soybean-Casein Digest [Wako Pure Chemical Industries, Ltd.]	18 h, 37°C
	*E*. *faecalis*	ATCC29212	Soybean-Casein Digest [Wako Pure Chemical Industries, Ltd.]	18 h, 37°C
	*E*. *coli*	ATCC25922	Soybean-Casein Digest [Wako Pure Chemical Industries, Ltd.]	18 h, 37°C
	*P*. *aeruginosa*	ATCC27853	Soybean-Casein Digest [Wako Pure Chemical Industries, Ltd.]	18 h, 37°C
	MRSA	Clinical isolate	Soybean-Casein Digest [Wako Pure Chemical Industries, Ltd.]	18 h, 37°C
	*M*. *terrae*	JCM12143	Middlebrook7H10 [Nippon Becton Dickinson Company, Ltd.]	8 days, 37°C
	*M*. *abscessus*	ATCC19977	Middlebrook7H10 [Nippon Becton Dickinson Company, Ltd.]	2 days, 37°C
	*B*. *cereus*	Clinical isolate	Soybean-Casein Digest [Wako Pure Chemical Industries, Ltd.]	1 day, 37°C
Fungi	*C*. *albicans*	NBRC1594	Sabouraud [Eiken Chemical Co., Ltd.]	2 days, Room temp.
	*T*. *mentagrophytes*	Clinical isolate	Sabouraud [Eiken Chemical Co., Ltd.]	4 days, Room temp.
	*A*. *niger*	NBRC105649	Sabouraud [Eiken Chemical Co., Ltd.]	2 days, Room temp.

### Setup of virus inactivation by multigas plasma jet

The viral inactivation portion of this study was conducted using the nonenveloped RNA viruses coxsackie virus A7 (CVA7) and feline calicivirus F9 (FCV/F9) with Vero cells (DSIU002, *Cercopithecus aethiops* kidney origin; DS Pharma Biomedical, Tokyo, Japan) for CVA7 and Crandell–Rees feline kidney (CRFK) cells (JCRB9035, cat kidney origin; Japanese Collection of Research Bioresources Cell Bank) for FCV/F9 as hosts [15]. Vero cells were cultured in Eagle’s minimal essential medium (EMEM; Nissui Pharmaceutical, Co. Ltd.) supplemented with 5% fetal bovine serum (FBS; CELLect, MP Biomedicaks LLC, Cedex, France). CRFK cells were cultured in EMEM with nonessential amino acids (NEAA; GIBCO, Grand Island, NY, USA) and 10% FBS. All susceptible cells were further cultured in EMEM supplemented with 1.0%–0.5% FBS for 2–3 days. After confirming degeneration, the cells were lysed by centrifugation at 3000 rpm for 20 min. Furthermore, the supernatant, as a viral preparation, was collected and stored at −80°C until assayed [15].

The viral suspensions were divided into 200 μl aliquots that were subsequently treated with nitrogen plasma and carbon dioxide plasma, as shown in Fig 1. After plasma treatment, 10-fold serial dilutions of the viral suspensions were prepared, which were then incubated in 96 well plates containing appropriate host cells. For viral infection, host cells were incubated in 100 μl of EMEM supplemented with 1% FBS at 37°C in a 5% CO_2_ atmosphere. After incubation for the specified time, to measure virucidal activity, the cytopathic effect (CPE) and 50% tissue culture infective dose (TCID_50_ 10 μl^−1^) of plasma-treated CVA7 and FCV/F9 were determined (*n* = 12).

### Identification of radical scavengers that effectively inactivate bacteria

To cancel out the inactivation effect of reactive species, the inactivation effects of individual radical scavengers, which react with various reactive species, were investigated. If the inactivation effect is reduced by adding a radical scavenger, the factor of the reactive species can be deduced. In this study, 10 mM of dimethyl sulfoxide (DMSO; Nacalai Tesque, Inc., Kyoto, Japan) as an OH radical scavenger [16], 10 mM of sodium azide (Wako Pure Chemical Industries, Ltd.) as a singlet oxygen scavenger [17], 300 U/ml of superoxide dismutase (SOD; Wako Pure Chemical Industries, Ltd.) as a superoxide scavenger [18], and 300 U/ml of catalase (Sigma-Aldrich Japan K.K., Tokyo, Japan) as an H_2_O_2_ scavenger [19] were prepared in 200 μl of PBS containing >10^6^ of *P*. *aeruginosa*. The suspensions were treated with carbon dioxide plasma and nitrogen plasma and the surviving bacteria were measured by counting of CFUs (*n* = 3).

## Results

### Inactivation of general bacteria by various gas plasmas

**Fig 2:**
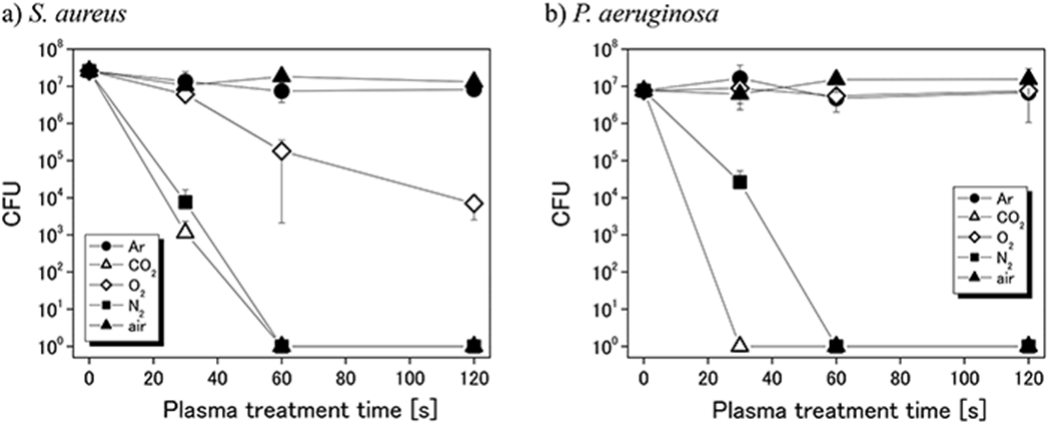
Bacterial inactivation in PBS(−) by various gas plasmas: (a) *S*. *aureus*; (b) *P*. *aeruginosa*.

To confirm bacterial inactivation, suspensions of *S*. *aureus* and *P*. *aeruginosa* were treated with the five gas plasmas and the effects were assessed by counting CFUs. It is found that both bacteria maintain CFUs even with the all gas flow treatment without discharge for 120 s. As shown in Fig 2, in case of *S*. *aureus* inactivation, carbon dioxide plasma and nitrogen plasma inactivated >6 log bacteria within 60 s, while oxygen plasma inactivated 3 log bacteria at 120 s. Mock air plasma and argon plasma inactivated <1 log bacteria at 120 s. In case of *P*. *aeruginosa* inactivation, carbon dioxide plasma and nitrogen plasma inactivated >6 log bacteria within 60 s. On the other hand, the bacterial inactivation effect of the other gas plasmas was <1 log. These results indicate high bacterial inactivation effects of carbon dioxide plasma and nitrogen plasma on both bacteria.

### Inactivation of viruses, fungi, acid-fast bacilli, and spore-producing bacteria

Tolerance to disinfection varies among microorganisms. Therefore, the treatment time for bacteria and viruses that exhibited a reduction of >6 log was investigated using carbon dioxide plasma and nitrogen plasma. As shown in Table 3, carbon dioxide plasma inactivated *E*. *coli*, *P*. *aeruginosa*, *E*. *faecalis*, *S*. *aureus*, and MRSA by >6 log within 1 min and by >6 log for *C*. *albicans* and FCV/F9 within 5 min, respectively. The other experimental microorganisms, *A*. *niger*, *T*. *mentagrophytes*, *M*. *abscessus*, *M*. *terrae*, *B*. *cereus* spores, and *Coxsackie virus*, were inactivated by <2 log at 10 min. On the other hand, nitrogen plasma inactivated all bacteria and viruses by >6 log. Regarding treatment time, effects were observed in *E*. *coli*, *P*. *aeruginosa*, *E*. *faecalis*, *S*. *aureus*, MRSA, *C*. *albicans*, and *M*. *abscessus* within 1 min, FCV/F9 within 3 min, *T*. *mentagrophytes*, *M*. *terrae*, and *Coxsackie virus* within 10 min, and *B*. *cereus* spores within 15 min. These results suggest that nitrogen plasma had a greater inactivation effect than carbon dioxide plasma in terms of wide microbicidal spectrum.

**Table 3:** Treatment times for plasmas that exhibited >6 log reduction of microorganisms.

Microorganism		>6 log reduction time (min)	
		CO_2_ plasma	N_2_ plasma
Gram-negative bacteria	*E*. *coli*	0.5	1
	*P*. *aeruginosa*	0.5	1
Gram-positive bacteria	*E*. *faecalis*	1	1
	*S*. *aureus*	1	1
	MRSA	1	0.5
	*M*. *abscessus*	2 log reduction in 10 min	1
	*M*. *terrae*	1 log reduction in 10 min	10
	*B*. *cereus* spore	>1 log reduction in 15 min	15
Fungi	*C*. *albicans*	5	1
	*A*. *niger*	>1 log reduction in 10 min	5
	*T*. *mentagrophytes*	2 log reduction in 10 min	10
Nonenveloped viruses	*Feline calicivirus*	5	3
	*Coxsackie virus*	2 log reduction in 10 min	10

### Effect of radical scavengers on plasma inactivation

**Fig 3:**
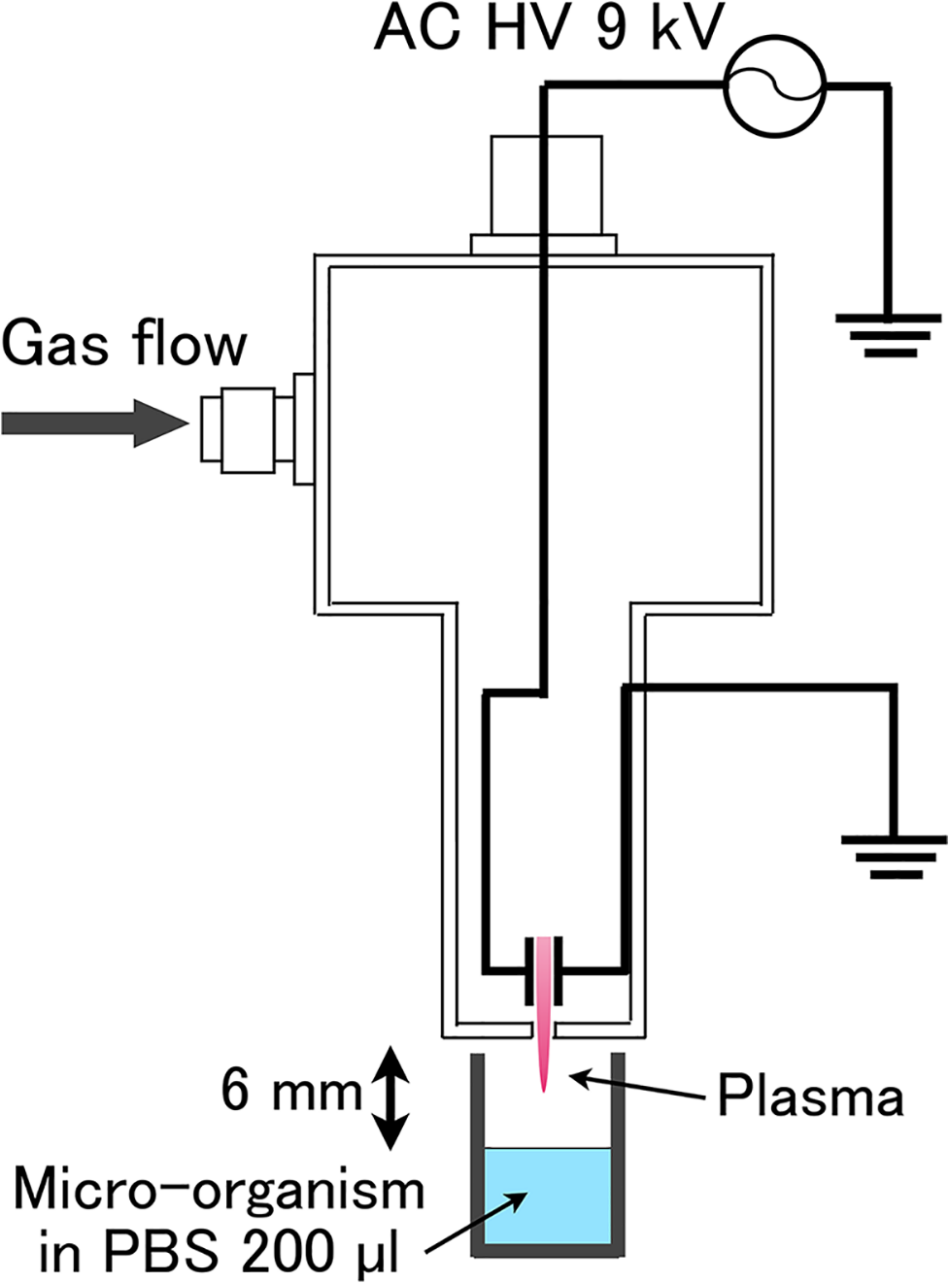
Inactivation effects of nitrogen plasma and carbon dioxide plasma on *P*. *aeruginosa* suspension including each radical scavenger. (Treatment time, 60 s; initial bacteria concentration, 5.4 × 10^7^ CFU). SOD was used as a superoxide scavenger, catalase as a H_2_O_2_ scavenger, NaN_3_ as a singlet oxygen scavenger, and DMSO as an OH radical scavenger.

Each radical scavenger was added to a suspension of *P*. *aeruginosa* and the bacterial inactivation effects of nitrogen plasma and carbon dioxide plasma were investigated. As shown in Fig 3, both plasmas inactivated *P*. *aeruginosa* by >6 log without radical scavengers. The addition of the OH radical scavenger (DMSO) suppressed the inactivation effects toward both plasmas. On the other hand, the singlet oxygen scavenger (NaN_3_) suppressed the bacterial inactivation effect of carbon dioxide, whereas nitrogen plasma inactivated *P*. *aeruginosa* by >6 log. Moreover, the bacterial suspensions including the H_2_O_2_ scavenger (catalase) and superoxide scavenger (SOD) were inactivated by >6 log with both plasmas. These results suggest that the OH and singlet oxygen radicals caused bacterial inactivation, whereas superoxide and H_2_O_2_ have minimal effects on bacterial inactivation.

## Discussion

In this study, the pH of bacterial suspensions was fixed at 7.3 – 7.5 with PBS(-) and we evaluated the effects of reactive species on bacterial inactivation among five plasma gas species using a multigas plasma jet and found that nitrogen plasma and carbon dioxide plasma performed most effectively. In addition, these effects were suppressed by the addition of the OH and singlet oxygen radical scavengers, although the effects remained unchanged by the addition of either the superoxide or H_2_O_2_ scavengers. Regarding the production of reactive species by carbon dioxide plasma, it appears that atomic oxygen and water generate OH and singlet oxygen radicals in accordance with Formulas (1) and (2) [16], [20], which suggests that both reactive species can inactivate bacteria [21].

$$O\;+\;H_{2}O\;\to \;2HO•$$ (1)

$$2O\;\to \;{}^{1}O_{2}$$ (2)

Regarding the production of reactive species by nitrogen plasma, it appears that atomic nitrogen and water generate OH radicals in accordance to Formula (3) [22], and singlet oxygen is generated secondarily as illustrated in Formula (4) [16]. Therefore, a significant amount of OH radicals was generated compared to production of the singlet oxygen radical, suggesting that the OH radical was mainly responsible for bacterial inactivation [21].

$$2N\;+\;2H_{2}O\;\to \;N_{2}\;+\;2HO•\;+\;2H•$$ (3)

$$HO•\;+\;O_{2}\;\to \;\left[HOOO•\right]*\to \;HO•\;+\;{}^{1}O_{2}\;\;\;*intermediate$$ (4)

On the other hand, the bacterial inactivation effects of the oxygen plasma and mock air plasma were lower than that of the carbon dioxide plasma and nitrogen plasma, which suggested that the amounts of singlet oxygen and OH radicals produced by the oxygen plasma and mock air plasma were lower than those produced by the carbon dioxide and nitrogen plasmas [23]. Regarding oxygen plasma, it appears that singlet oxygen radicals generated in the atmosphere react with triplet oxygen to produce ozone, which has a lower oxidation capacity than the singlet oxygen radical, as shown in Formula (5) [4]. Hence oxygen plasma yields higher concentrations of triplet oxygen radicals than carbon dioxide plasma. Regarding the mock air plasma, because of the reactions of the atomic oxygen and nitrogen generated, nitrite and nitrate were mainly produced instead of singlet oxygen and OH radicals [16]. These findings were confirmed based on previous measurements of reactive species [23].

$$O\;+\;O_{2}\;+\;M\;\to \;O_{3}\;+M$$ (5)

As shown in Table 2, plasma treatment of various microorganisms showed that bacterial inactivation by the nitrogen plasma was best among the five gas species. In our previous reports, we demonstrated that among these five gas species, nitrogen plasma generated the greatest amount of OH radicals and carbon dioxide generated the largest amount of singlet oxygen radicals [23]. These findings, in conjunction with those of the present study, indicate that the OH radical is more effective for bacterial inactivation than singlet oxygen radicals. Furthermore, the time required to inactivate bacteria is dependent on the microorganism species, e.g., the plasma-inactivated general bacteria (*E*. *coli*, *P*. *aeruginosa*, *E*. *faecalis*, *S*. *aureus*, and MRSA) by 6 log within 1 min; however, it took approximately 15 min to inactivate spore bacteria (*B*. *cereus*) by the same amount.

As a possible explanation of these results, we believe that the cell wall structures of general bacteria were more difficult to mechanically rupture [24]. However, because details of the involved mechanisms remain unclear, future studies are needed to determine the effects of reactive species on cellular integrity. Using our multigas plasma jet, we identified reactive species that have the capacity to inactivate bacteria, thus continued research to define site-specific damage to the microorganism is warranted.

In conclusion, our novel multigas plasma jet system effectively inactivated various bacteria, fungi, and viruses; therefore, should be considered for medical applications, such as sterilization of surgical instruments and disinfection of wounds.
