# Correction: Microbial Inactivation in the Liquid Phase Induced by Multigas Plasma Jet

**DOI:** 10.1371/journal.pone.0135546

**Published:** 2015-08-07

**Authors:** Toshihiro Takamatsu, Kodai Uehara, Yota Sasaki, Miyahara Hidekazu, Yuriko Matsumura, Atsuo Iwasawa, Norihiko Ito, Masahiro Kohno, Takeshi Azuma, Akitoshi Okino

The images for Figs 1 and 3 are incorrectly switched. The image that appears as Fig 1 should be Fig 3, and the image that appears as Fig 3 should be Fig 1. The figure captions appear in the correct order. Please see the correct Figs 1 and 3 here.

**Fig 1:**
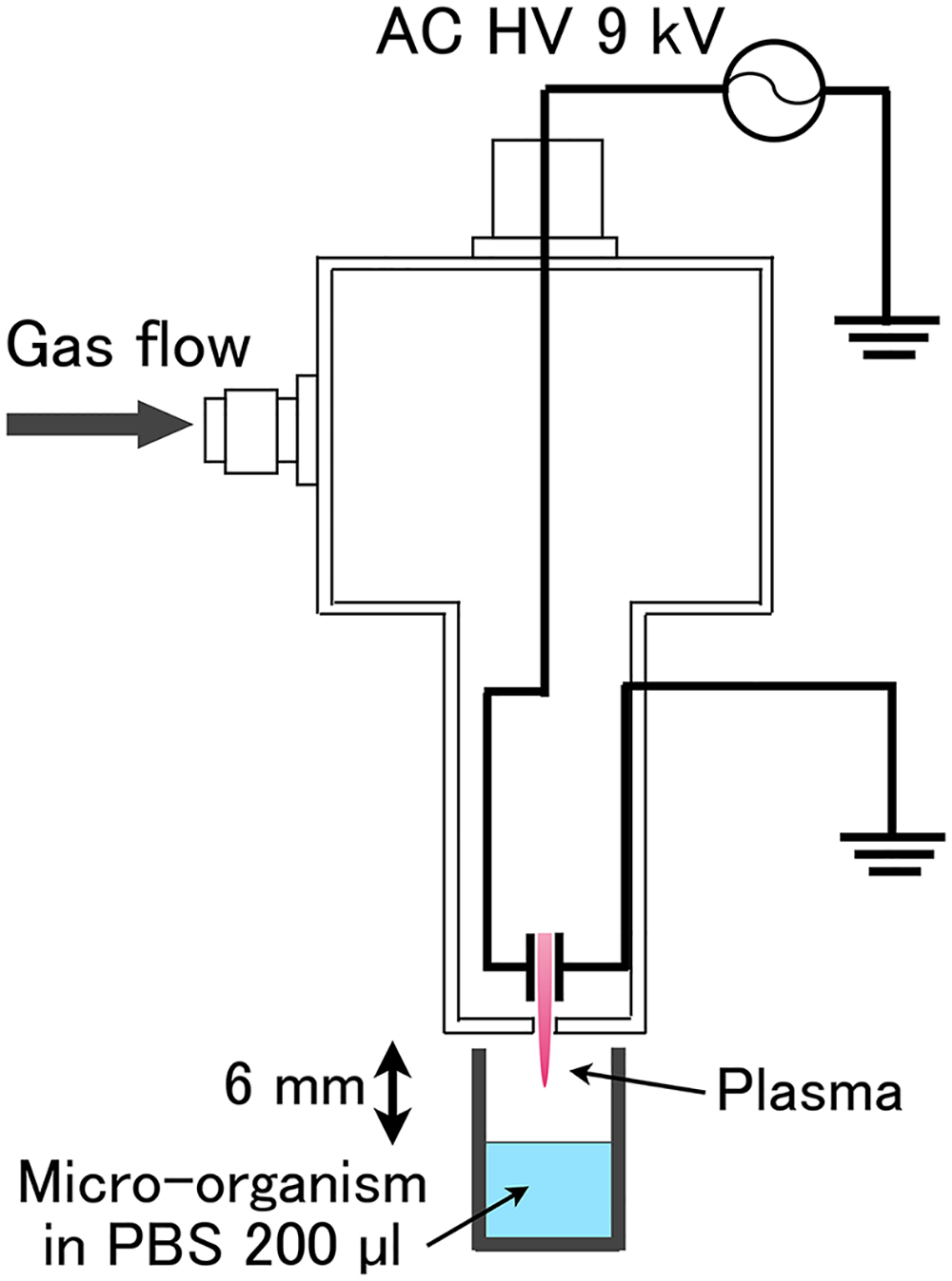
A schematic of the plasma treatment system using a multigas plasma jet source.

**Fig 3:**
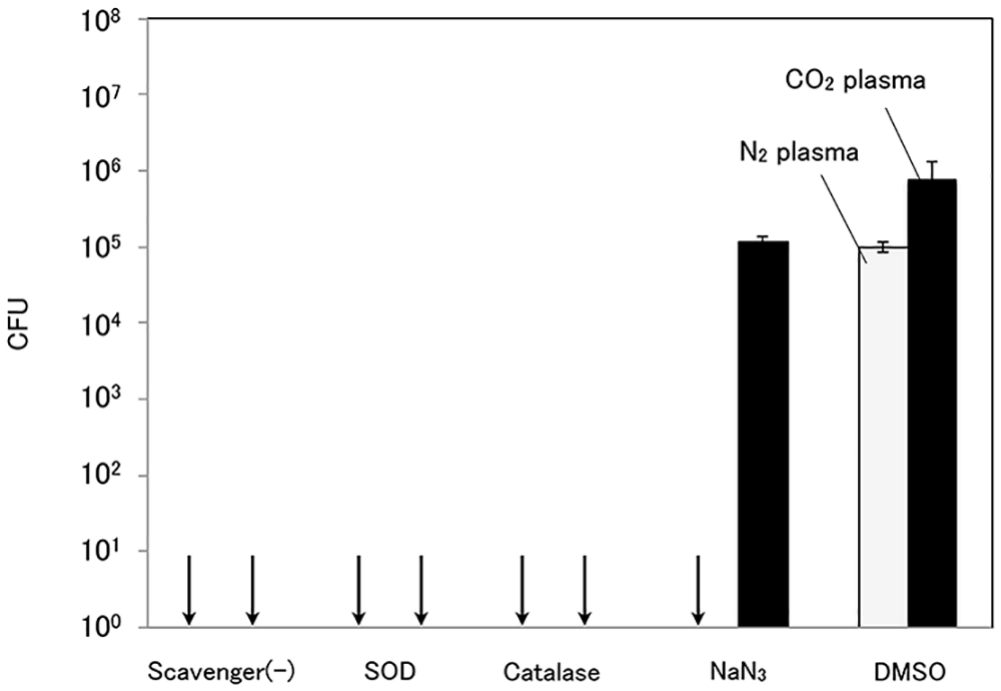
Inactivation effects of nitrogen plasma and carbon dioxide plasma on *P*. *aeruginosa* suspension including each radical scavenger. (Treatment time, 60 s; initial bacteria concentration, 5.4 × 10^7^ CFU). SOD was used as a superoxide scavenger, catalase as a H_2_O_2_ scavenger, NaN_3_ as a singlet oxygen scavenger, and DMSO as an OH radical scavenger.
